# Can ACE2 Receptor Polymorphism Predict Species Susceptibility to SARS-CoV-2?

**DOI:** 10.3389/fpubh.2020.608765

**Published:** 2021-02-10

**Authors:** Christian A. Devaux, Lucile Pinault, Ikram Omar Osman, Didier Raoult

**Affiliations:** ^1^Aix-Marseille Université, IRD, APHM, MEPHI, IHU–Méditerranée Infection, Marseille, France; ^2^CNRS, Marseille, France; ^3^Fondation IHU–Méditerranée Infection, Marseille, France

**Keywords:** COVID-19, SARS-CoV-2, coronavirus, ACE2, *in silico* analyses

## Abstract

A novel severe acute respiratory syndrome coronavirus, SARS-CoV-2, emerged in China in December 2019 and spread worldwide, causing more than 1.3 million deaths in 11 months. Similar to the human SARS-CoV, SARS-CoV-2 shares strong sequence homologies with a sarbecovirus circulating in *Rhinolophus affinis* bats. Because bats are expected to be able to transmit their coronaviruses to intermediate animal hosts that in turn are a source of viruses able to cross species barriers and infect humans (so-called spillover model), the identification of an intermediate animal reservoir was the subject of intense researches. It was claimed that a reptile (*Ophiophagus hannah*) was the intermediate host. This hypothesis was quickly ruled out and replaced by the pangolin (*Manis javanica*) hypothesis. Yet, pangolin was also recently exonerated from SARS-CoV-2 transmission to humans, leaving other animal species as presumed guilty. Guided by the spillover model, several laboratories investigated *in silico* the species polymorphism of the angiotensin I converting enzyme 2 (ACE2) to find the best fits with the SARS-CoV-2 spike receptor-binding site. Following the same strategy, we used multi-sequence alignment, 3-D structure analysis, and electrostatic potential surface generation of ACE2 variants to predict their binding capacity to SARS-CoV-2. We report evidence that such simple *in silico* investigation is a powerful tool to quickly screen which species are potentially susceptible to SARS-CoV-2. However, possible receptor binding does not necessarily lead to successful replication in host. Therefore, we also discuss here the limitations of these *in silico* approaches in our quest on the origins of COVID-19 pandemic.

## Introduction

The recent emergence of SARS-CoV-2, responsible for a respiratory disease named COVID-19 (Coronavirus Disease-2019), threatens public health (1–4). SARS-CoV-2 is responsible for respiratory infections, frequently asymptomatic but sometimes progresses to pneumonia, which, in its most severe forms can lead to death. SARS-CoV-2 is spreading very rapidly worldwide, and since WHO has declared COVID-19 as pandemic, about 54.5 million people have been infected worldwide (https://coronavirus.jhu.edu/map.html; 16 November, 2020). The case fatality rate of COVID-19 (estimated about 3.29%) increases with age and the existence of underlying diseases (2, 3, 5). Recently, Fang et al. (6) reported that the most distinctive comorbidities in patients who died from COVID-19 are hypertension, coronary heart diseases, cerebrovascular diseases, and diabetes. Soon after the characterization of SARS-CoV-2, it was demonstrated that this virus uses the angiotensin I converting enzyme 2 (ACE2) expressed on pneumocytes to enter human cells (7, 8). Several recently published papers reported the SARS-CoV-2 entry into target cells through interactions with ACE2 and serine protease TMPRSS2 priming, as well as the three-dimensional (3-D) structures involved in the interactions between the viral spike (S) protein and ACE2 (9–13). The polymorphism of ACE2 in human populations was recently documented, suggesting that these allelic differences could translate into differences in susceptibility to SARS-CoV-2 infection (14, 15). Insofar as the physiological function of ACE2 is to cleave angiotensin II into angiotensin (1–7), SARS-CoV-2 infection could cause a dysregulation of this peptidase leading to risk of malfunction of the Renin–Angiotensin–Aldosterone pathway (16).

SARS-CoV-2 is the 7th human coronavirus (HCoV) reported to date. Previously, the first HCoVs described back in the 1960s were the HCoV-229E (*Alphacoronavirus*) and HCoV-OC43 (*Betacoronavirus* lineage 2a), two agents of common winter cold. In 2003, the coronaviruses gained in notoriety with the emergence in Asia of SARS-CoV (*Betacoronavirus* lineage 2b), proven responsible for a severe acute respiratory syndrome (SARS) in humans with a case fatality rate of 9.6% (17). Within the next couple of years, the HCoV-NL63 (*Alphacoronavirus* lineage 1b) and HCoV-HKU1 (*Betacoronavirus* lineage 2a) were discovered. The HCoV-HKU1 was discovered in Hong Kong. The case fatality rate of the four HCoVs responsible for common winter cold was estimated to be 0.5–1.5% (18–20). In contrast to SARS-CoV and HCoV-HKU1 that emerged in Southeast Asia suggesting that this region is probably a hotspot for coronavirus emergence, the Middle East Respiratory Syndrome (MERS), caused by the MERS-CoV (*Betacoronavirus* lineage 2c), was reported in Saudi Arabia in 2012. This epidemic was characterized by an extremely high case fatality rate of 34.7% (21). The last coronavirus known to infect humans, SARS-CoV-2 (*Betacoronavirus* lineage 2b/Sarbecovirus), emerged in China in 2019 and shows 79.5% nucleotide identity with SARS-CoV (22). It is interesting to highlight that HCoV-NL63, SARS-CoV, and SARS-CoV-2 spike proteins bind ACE2, indicating that several members of the coronavirus family have developed a preferential tropism for this receptor to enter target cells [(23, 24); Table 1].

**Table 1:** Viral tropism.

**Receptor**	**Virus**	**Primary site of disease**	**Other organs**
ACE2	SARS-CoV-1	Lower respiratory tract	Multi-organ failure
	SARS-CoV-2	Lower respiratory tract	Multi-organ failure
	HCoV-NL63	Upper respiratory tract	–
DPP4/CD26	MERS-CoV	Lower respiratory tract	Myocarditis, renal failure
CD13	HCoV-229E	Upper respiratory tract	Gastrointestinal
HLA Class I	HCoV-OC43	Upper respiratory tract	Gastrointestinal
	HCoV-HKU1	Upper respiratory tract	Gastrointestinal

As soon as this new SARS-CoV-2 was discovered, many studies were initiated to understand the viral infection mechanisms and to clarify its origin. Consequently, the search for animal hosts was considered of high urgency for the control of COVID-19. The very first investigations focused on bats (order *Chiroptera*), which are considered a reservoir for coronaviruses (CoV) and can be a source of epizootic and zoonosis (25–27). With 1,230 species, bats have the second highest number of species (after rodents) in the mammal world. They inhabit a multitude of ecological niches and carry a huge number of zoonotic viruses worldwide (28, 29). The probability for CoV to cross species barrier is higher in Southeast Asia where bats are sold in wildlife wet markets. Different species of *Rhinolophus* bats in China carry genetically diverse SARS-like coronaviruses, some of which are direct ancestors of SARS-CoV and SARS-CoV-2 (30, 31). Based on our knowledge of coronaviruses circulating in Chinese bats, it is not a surprise that SARS-CoV-2 was also considered to have originated from *Rhinolophus* bats. This turned out to be confirmed by elegant results showing that SARS-CoV-2 shares 96.2% identity with the BatCoV RaTG13 strain from *Rhinolophus affinis* (22). Then, many laboratories started looking after an intermediate animal host. The snake (*Ophiophagus hannah*) and the pangolin (*Manis javanica*) were claimed to be intermediate hosts. The snake hypothesis was quickly ruled out (32). Although the pangolin hypothesis was the mainstream, it was also recently excluded (33, 34). At the same time, other species were singled out. To quickly study a large number of potential targets without having to grow virus on cells or infect animals with SARS-CoV-2, *in silico* approaches seemed to be a quite appropriate strategy since the three-dimensional structure of the S1 protein of SARS-CoV-2 was resolved, allowing the specification of the amino acids important for binding to ACE2 (9–13, 35). The knowledge previously accumulated on the interaction between ACE2 and the SAR-CoV spike was also of great value (23, 36, 37). Interestingly, K353 and N90 in ACE2 are essential for infection likely due to their effect on the conformation of the α-helix 1 of the receptor.

We revisited here the predicted binding properties between the viral S protein of SARS-CoV-2 and its ACE2 receptor, using *in silico* analysis based on alignment of receptor protein sequences from different species and structural modeling of ACE2 receptors. We found a good match between the *in silico* predictions of virus tropism and the species already considered to be possible intermediates between bats and humans for transmission of SARS-CoV-2. We report that positions K31 and Y41 in the α1 ridge, N82 and N90 in the loop, and α3 and K353 in loop and β5 are those that must be examined in order to predict the possibility of a species to become infected by SARS-CoV-2. In agreement with previous reports suggesting that exchange N90T destroys a major N-glycosylation site in ACE2 (9, 10, 38), we confirm that N90 is likely a critical position in ACE2 for SARS-CoV-2 binding. The analysis of electrostatic potential surface generation of ACE2 variants highlight minor differences in surface charges for mouse and frog sequence insertions compatible with lower susceptibility of these species to SARS-CoV-2. Finally, the broad spectrum of potentially susceptible species argues in favor of the circulation model (33) rather than in favor of the spillover model (39).

## Materials and Methods

### ACE2 Protein Sequence

ACE2 protein sequences from the NCBI reference sequence database: *Rousettus leschenaultii* (GenBank: ADJ19219.1), *Rousettus leschenaultii* (GenBank: BAF50705.1), *Rousettus aegyptiacus* (NCBI Reference Sequence: XP_015974412.1), *Pteropus alecto* (NCBI Reference Sequence: XP_006911709.1), *Pteropus vampyrus* (NCBI Reference Sequence: XP_011361275.1), *Phyllostomus discolor* (NCBI Reference Sequence: XP_028378317.1), *Desmodus rotundus* (NCBI Reference Sequence: XP_024425698.1), *Miniopterus natalensis* (NCBI Reference Sequence: XP_016058453.1), *Pipistrellus abramus* (GenBank: ACT66266.1), *Eptesicus fuscus* (NCBI Reference Sequence: XP_008153150.1), *Myotis davidii* (NCBI Reference Sequence: XP_015426918.1), *Myotis lucifugus* (NCBI Reference Sequence: XP_023609438.1), *Myotis brandtii* (NCBI Reference Sequence: XP_014399780.1), *Hipposideros armiger* (NCBI Reference Sequence: XP_019522936.1), *Rhinolophus ferrumequinum* (GenBank: ADN93470.1), *Rhinolophus pearsonii* (GenBank: ABU54053.1), *Rhinolophus sinicus* (GenBank: AGZ48803.1), *Rhinolophus pusillus* (GenBank: ADN93477.1), *Rhinolophus macrotis* (GenBank: ADN93471.1), *Homo sapiens* (GenBank: BAB40370.1), *Macaca mulatta* (NCBI Reference Sequence: NP_001129168.1), *Paguma larvata* (GenBank: AAX63775.1), *Felis catus* (GenBank: AAX59005.1), *Mustela putorius furo* (NCBI Reference Sequence: NP_001297119.1), *Sus scrofa domestic* (GenBank: ASK12083.1), *Sus scrofa* (NCBI Reference Sequence: NP_001116542.1), *Rhinolophus sinicus* (GenBank: AGZ48803.1), *Manis javanica* (NCBI Reference Sequence: XP_017505752.1), *Mus musculus* (NCBI Reference Sequence: NP_081562.2), *Rattus rattus* (NCBI Reference Sequence: XP_032746145.1), *Gallus gallus* (GenBank: QEQ50331.1), *Pelodiscus sinensis* (NCBI Reference Sequence: XP_006122891.1), *Xenopus tropicalis* (NCBI Reference Sequence: XP_002938293.2), and *Ophiophagus hannah* (GenBank: ETE61880.1).

### Comparison of Sequences

All ACE2 sequences were compared using the Clustal Omega multiple sequence alignment (EMBL-EBI bioinformatic tool; Copyright © EMBL 2020) (https://www.ebi.ac.uk/Tools/msa/clustalo/). The simple Unweighted Pair Group Method with Arithmetic Mean (UPGMA) algorithm of hierarchical clustering available under the Clustal Omega tool was used to produce rooted dendrogram (pairwise similarity matrix to construct a phylogenetic tree).

### 3-D Analysis and Electrostatic Potential Surface Generation

The 3-D structure of ACE2 was retrieved according to the published data [PDB: 6M1D; (11)]. Three amino acid segments (30–41, 82–93, and 353–358) from *R. sinicus, M. musculus*, and *X. tropicalis* ACE2 proteins were inserted into a human ACE2 backbone sequence to determine whether or not these substitutions may change the 3-D structure of ACE2. Protein modeling for these chimeric sequences was performed using the Phyre2 server (40). The PyMOL 1.8.0 software (https://sourceforge.net/projects/pymol/files/pymol/1.8/) and the Adaptative Poisson-Boltzmann Solver (APBS) tools plugin (https://pymolwiki.org/index.php/APBS) were used to generate electrostatic potential surfaces of the human ACE2 and its modified chimeric versions. The red color indicates an excess of negative charges near the surface and the blue color arises from a positively charged surface.

## Results

### ACE2 Receptor Polymorphism Among Species

**Figure 1:**
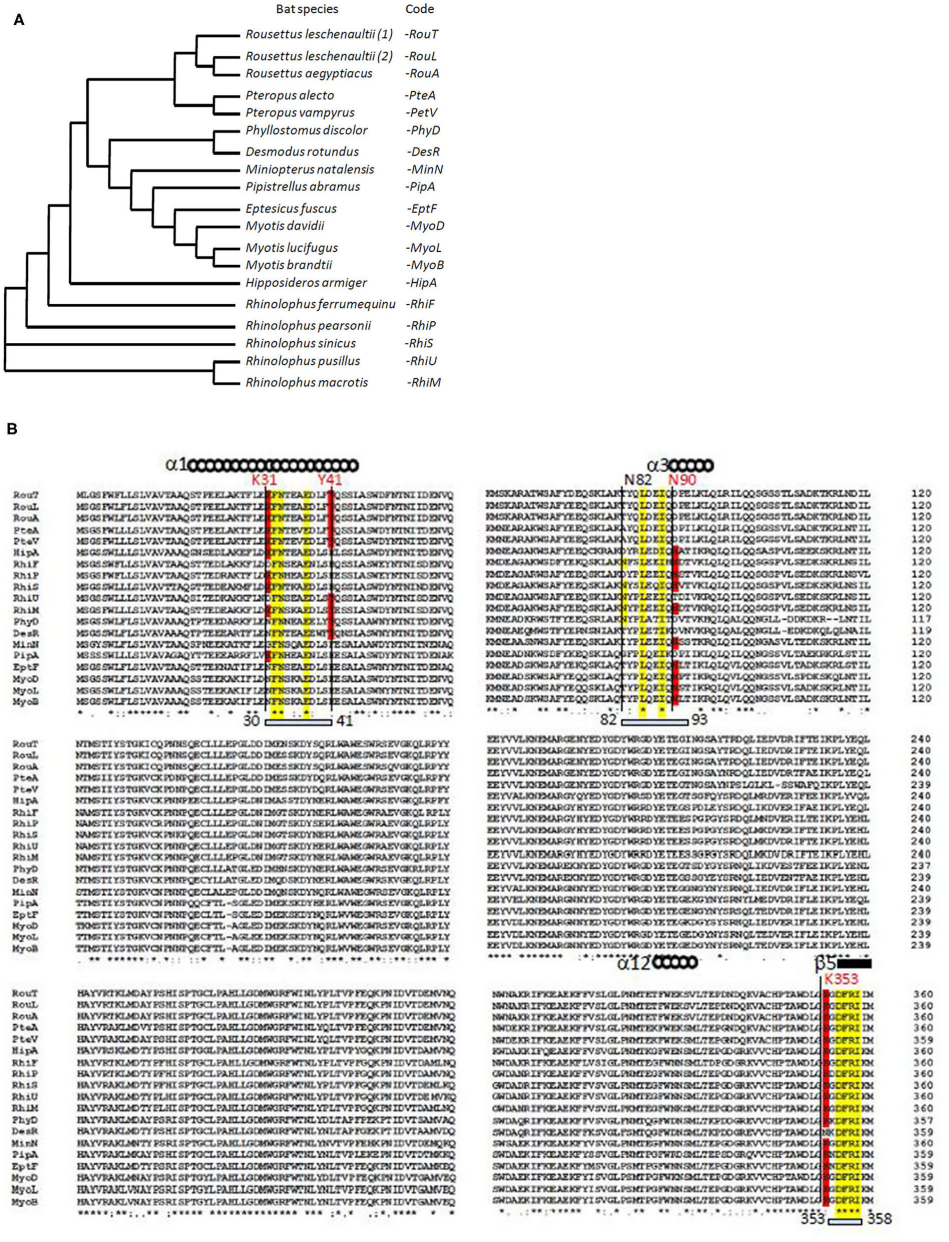
Bat ACE2 sequence alignment. The ACE2 protein sequences from 18 species of bats were obtained from the NCBI reference sequence database: *Rousettus leschenaultii, Rousettus aegyptiacus, Pteropus alecto, Pteropus vampyrus, Phyllostomus discolor, Desmodus rotundus, Miniopterus natalensis, Pipistrellus abramus, Eptesicus fuscus, Myotis davidii, Myotis lucifugus, Myotis brandtii, Hipposideros armiger, Rhinolophus ferrumequinum, Rhinolophus pearsonii, Rhinolophus sinicus, Rhinolophus pusillus*, and *Rhinolophus macrotis*. Clustal Omega multiple sequence alignment (EMBL-EBI bioinformatic tool; Copyright © EMBL 2020) was used to compare the ACE2 protein sequences of these mammals considered at the origin of human coronaviruses. **(A)** Phylogenetic tree of bat ACE2 sequences built using the Clustal Omega multiple sequence alignment program and the UPGMA algorithm. The short code is used in **(B)**. **(B)** Sequences alignment of bat ACE2 N-terminal (amino acids 1–360 of 805) protein sequences. Some of the amino acids important for viral tropism are in red (previous studies showed that residues 31 and 41 and regions 82–84 and 353–357 are important for viral spike binding). Within the regions considered important for the interaction with the spike of SARS-CoV-2, the conserved amino acids are in yellow.

Using multiple sequence alignment, we first compared the ACE2 sequences of 18 bat species. We found that the variant ACE2 proteins perfectly grouped in the dendrogram according to the subspecies of bats (Figure 1A). When we studied the multiple sequence alignments of ACE2 from bats and examined the regions predicted by crystallography to be the regions of contact with the S1 spike of the SARS-CoV-2 (Figure 1B), we observed significant differences between species. *Rhinolophus* bats appeared to be appropriate candidates for binding to SARS-CoV-2-related viruses, yet a species polymorphism was observed among the *Rhinolophus* (i.e., *R. sinicus* with K31, Y41H, N82, N90, and K353 and *R. ferrumequinum* with K31D, Y41H, N82, N90, K353). The K31D variant may possibly alter the binding of the SARS-CoV-2 spike to the ACE2 from *R. ferrumequinum*. Unfortunately, the ACE2 sequence of *R. affinis* hosting the closest relative to SAR-CoV-2, BatCoV-RaTG13, was not available from the database. The ACE2 sequences from other bat species living in various ecosystem and with various geographic distribution, show increased amino acids substitutions at positions considered to be required for viral S1 spike binding (e.g., *D. rotundus* with K31N, Y41, N82T, N90D, and K353N). It is worth noting that the three *Rousettus* and two *Pteropus* ACE2 proteins analyzed in this study were characterized by K31, Y41, and N82T (*Rousettus*) or N82A (*Pteropus*), N90D, and K353. We also found that the three ACE2 proteins from *Myotis* bats were characterized by K31N, Y41H, N82T, N90, and K353, suggesting that these species are unlikely to replicate SARS-CoV-2-like ancestor-related viruses.

### The Central Role Played by ACE2 for Interspecies Virus Spread

Unraveling which cellular receptors are used by SARS-CoV-2 for entry should provide insights into viral transmission among species. Before SARS-CoV-2, SARS-like CoV was previously found to circulate in Chinese horseshoe bats and to spread through wild Himalayan palm-civet sold as food in Chinese wildlife markets from Guangdong (41–43). SARS-CoV was also identified in weasels and raccoons in Chinese wet markets (37, 41, 43). Regarding SARS-CoV-2, the question remains how it got to humans. The hypothesis of pangolin (*M. javanica*) as an intermediate host for SARS-CoV-2 quickly became mainstream (32, 44). We recently demonstrated that pangolin is unlikely to be the intermediate host and that transmission to humans could just as easily have taken place via another animal (33).

**Figure 2:**
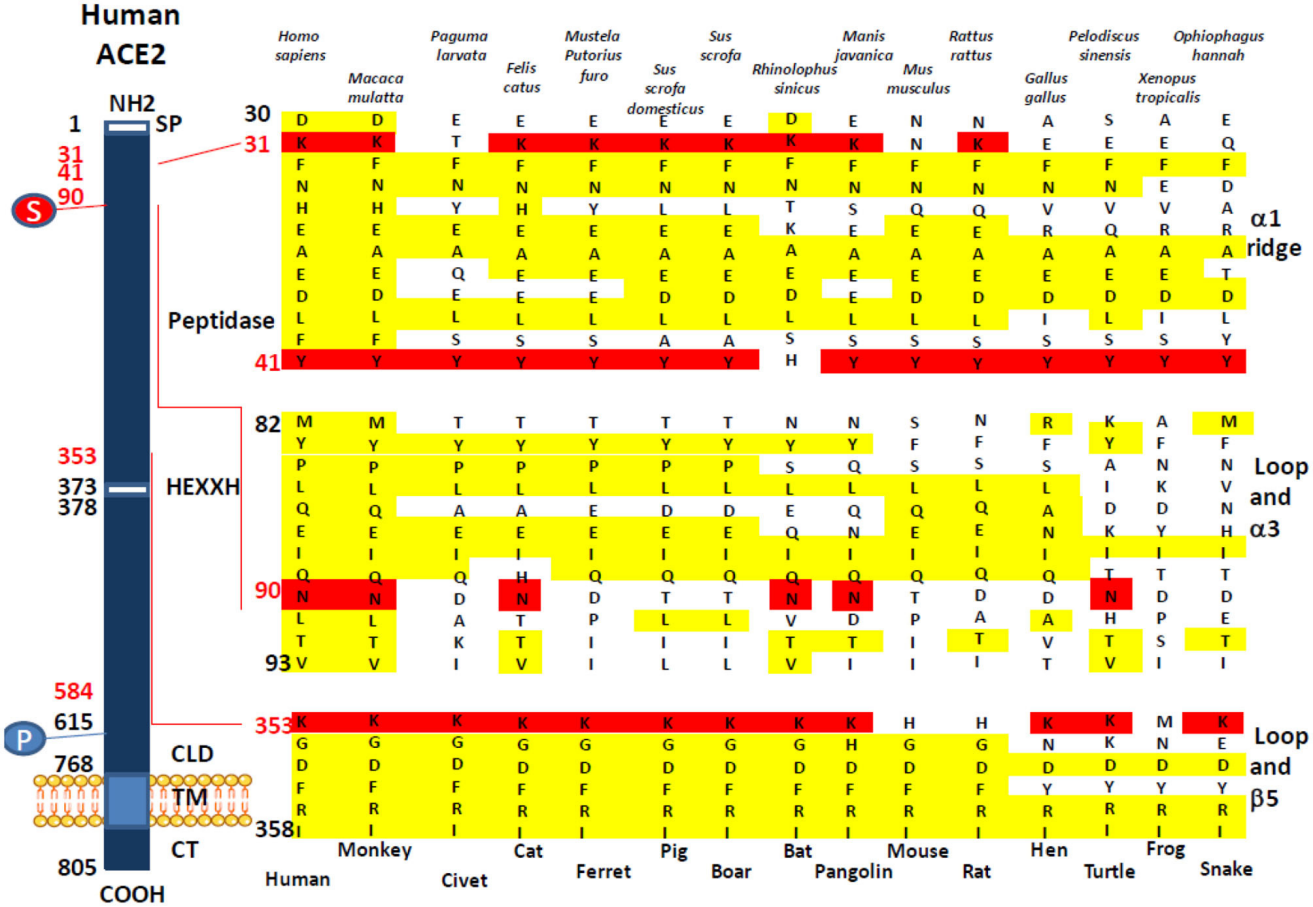
Comparison of the *Homo sapiens* ACE2 protein sequence and sequences from different mammals, birds, amphibians, and reptiles. A schematic representation of the cell surface human ACE2 molecule and its major domains is drawn on left side of the figure. The amino acid positions are in black. Some of the amino acids considered important for viral tropism are in red. S, sugar; P, phosphorylation. A comparison of ACE2 sequences from 15 different species using Clustal Omega multiple sequence alignment is shown in the right side of the figure. All sequences were numbered according to amino acid position on the *H. sapiens* ACE2 protein. All sequences were obtained from the NCBI reference sequence database. They include *Macaca mulatta* (monkey), *Paguma larvata* (palm civet), *Felis catus* (cat), *Mustela putorius furo* (ferret), *Sus scrofa domestic* (pig), *Sus scrofa* (boar), *Rhinolophus sinicus* (bat), *Manis javanica* (pangolin), *Mus musculus* (mouse), *Rattus rattus* (rat), *Gallus gallus* (hen), *Pelodiscus sinensis* (turtle), *Xenopus tropicalis* (frog), and *Ophiophagus hannah* (snake).

We investigated the amino acid substitutions in 14 species of mammals, birds, reptiles, and amphibians, expected to be possible intermediate hosts for SARS-CoV-2 (Figure 2). Beside positions K31, Y41, and K353 reported in several studies to have been playing a major role for SARS-CoV-2 spike binding to ACE2, our multisequence alignment suggested that species carrying an N90 are more likely to be susceptible to SARS-CoV-2 infection (it includes *H. sapiens, M. mulatta, F. catus, R. sinicus, M. javanica*, and *P. sinensis*) while others should be less susceptible to infection, except if the virus adapts to a second receptor for cellular binding and entry.

### Amino Acids K31, Y41, N90, and K353 in ACE2 Are Likely to Confer Susceptibility to SARS-CoV-2

**Figure 3:**
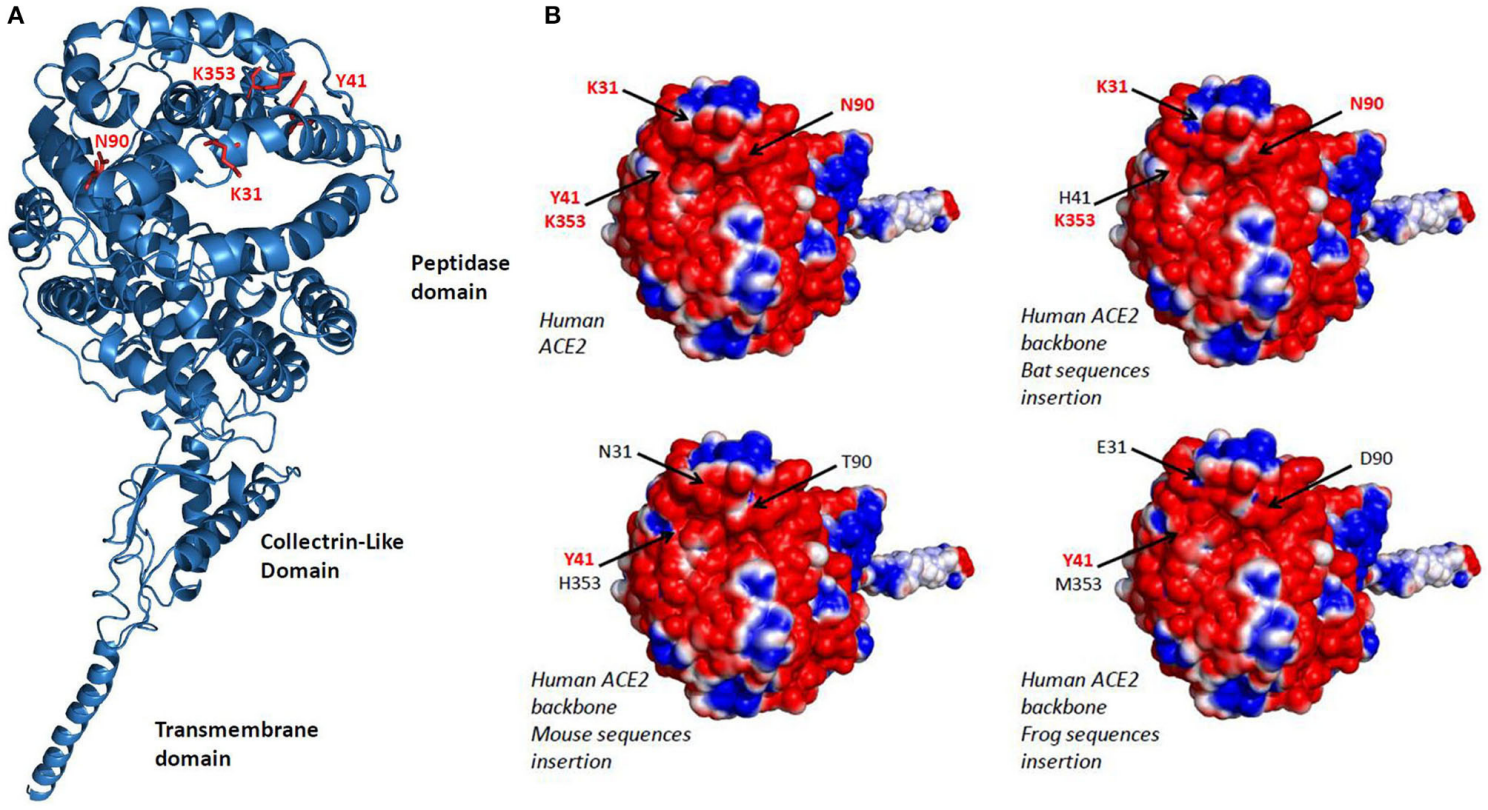
3-D model of ACE2. **(A)** Variant amino acids segments (30–41, 82–93, and 353–358) from *Rhinolophus sinicus, Mus musculus*, and *Xenopus tropicalis* species were superimposed on the *Homo sapiens* ACE2 3-D structure using the Phyre2 server. This model lacks the cytoplasmic tail of ACE2. **(B)** An electrostatic potential surface was generated using the PyMOL 1.8.0 software along with the APBS tool plugin. The upper left panel is a model of *H. sapiens* ACE2 extracellular region with its electrostatic potential distribution (red, acidic; white, neutral; blue, basic). The upper right and the two lower images represent simulation in which the α1 ridge, loop and α3, and loop and β5 (see Figure 2, right panel) sequences from *R. sinicus* (bat), *M. musculus* (mouse), and *X. tropicalis* (frog) were substituted to the corresponding human sequences in a *H. sapiens* ACE2 backbone. The locations of amino acids 31, 41, 90, and 353 are indicated by arrows.

The analysis of 3-D structures of different ACE2 with respect to the amino acids found in regions 30–41, 82–93, and 353–358 was studied after designing a backbone from the *H. sapiens* ACE2 in which the corresponding regions from *R. sinicus, M. musculus*, and *X. tropicalis* species were substituted to that from human. We found (Figure 3A) that these substitutions did not change the global 3-D structure of the molecule. However, when we analyzed the electrostatic potential surface of ACE2, more particularly in the regions 30–41, 82–93, and 353–358, we found that the substitution of those human ACE2 segments by the corresponding regions from *R. sinicus, M. musculus*, and *X. tropicalis* species slightly altered the electrostatic pattern of the molecule (Figure 3B). Indeed, in the region where amino acids Y41 and K353 are located in the human ACE2, when this region was substituted by sequences from mouse or frog origins, we observed a shift from neutral to basic electrostatic surface whereas the substitution for bat sequence did not change the electrostatic charge. The electrostatic surface was also different when the region containing K31 was substituted by that from bat or frog. These modifications are likely to be sufficient to reduce the interaction between SARS-CoV-2 spike and the variant ACE2.

## Discussion

Soon after the discovery of SARS-CoV-2, the cell surface exopeptidase ACE2 was found to serve as a viral receptor in human, and the first investigation of species susceptibility to this new virus demonstrated that SARS-CoV-2 is able to use Chinese horseshoe bat and swine but not mouse ACE2 to bind host cells (22). Since this pioneering work, several laboratories have intended to predict the utilizing capability by SARS-CoV-2 of ACE2 from different species using amino acid sequence comparisons aimed at identifying the possible intermediate hosts of SARS-CoV-2. This was made possible after published crystallographic analyses had determined which amino acids of ACE2 are essential for the attachment of the viral spike protein (9–11).

Our investigation suggests that SARS-CoV-like ancestral coronaviruses have adapted to the ACE2 receptor to replicate in bats. However, our analysis also suggests that probably not all bat species support SARS-CoV-like coronavirus ACE2 tropism. According to multisequence alignment, *Rhinolophus* bats appear to be appropriate candidates for ACE2 interaction with SARS-CoV-2-related viruses, yet a species polymorphism in ACE2 sequences is observed among the *Rhinolophus. R. sinicus* with K31, Y41H, N82, N90, and K353 is a good candidate for SARS-CoV-2-like virus capture whereas *R. ferrumequinum* with K31D, Y41H, N82, N90, and K353 can be predicted less susceptible to the virus binding. ACE2 sequences from other bat species show increased amino acid substitutions at positions considered required for viral spike binding (e.g., *D. rotundus* with K31N, Y41, N82T, N90D, and K353N). In species expressing variant ACE2 not suitable for virus binding, another surface receptor could serve as viral entry into cells, but such viruses will be less likely to cross species barriers using an ACE2 protein as receptor in an intermediate host species. This can support the hypothesis of a long bat and virus co-evolution with bat species that replicate ACE2-tropic viruses like SARS-CoV and other species that replicate CD26-tropic viruses like MERS-CoV.

In order that a SARS-CoV-2-like virus can leave bats to infect another susceptible host, the infected bat must come into contact with an animal expressing an ACE2 receptor adapted to SARS-CoV-2-like virus binding. In agreement with other studies (44–46), our *in silico* search for host species able to pass the SARS-CoV-2 to humans supports the hypothesis that species bearing K31 and K353 amino acids are more likely to bind SARS-CoV-2. For example, ACE2 from *M. javanica, M. putorius furo*, and *F. catus*, considered SARS-CoV-2-susceptible species, show K31 and K353 amino acids whereas *M. musculus*, which is considered a SARS-CoV-2-resistant species, shows a K31N and K353H variant. A Y41 also seems to be important, yet *R. sinicus* ACE2 expresses a Y41H variant. It may account for the requirement of an intermediate host before being able to infect humans. A position not particularly stressed out in several SARS-CoV-2 studies that appear important is N90. Indeed, the N90 that is found in *H. sapiens* final host and *R. sinicus* early host is also found in *M. mulatta, M. javanica, F. catus*, and *P. sinensis*, previously described susceptible to SARS-CoV-2 and possible intermediate hosts whereas N90D or N90T variants are found in the other species studied. This is also consistent with the earlier observation indicating that N90 was important for SARS-CoV and SARS-CoV-2 binding to ACE2 (9, 10, 23, 38). This may explain why N90 may be very important for infection of host cells. Among the various *in vitro* antiviral activities of chloroquine described to date, it has been suggested that this molecule could prevent the glycosylation of ACE2 (47, 48). We could hypothesize that chloroquine blocks the N-glycosylation at position 90 of the ACE2 sequence, thereby preventing the attachment of SARS-CoV-2 spike to the receptor. However, what is surprising is the sequence of the *P. larvata* with K31T, Y41, N82T, N90D, and K353, since palm civet has been considered as the intermediate host for SARS-CoV and suggested to also serve as a possible intermediate host for SARS-CoV-2 (45), whereas with the absence of K31, in addition to N82 and N90 (which are expected to be glycosylated, thereby favoring interaction with the viral spike), palm civet appears to be an animal unlikely to be infected through ACE2. This discrepancy, also noticed by Wan et al. (35), should be further explored. Another surprising result is the absence of an N90 glycosylation site in the ACE2 from *M. putorius furo* since ferrets are now known to be susceptible to infection with SARS-CoV-2 (49–52). It would be interesting to study the affinity of the SARS-CoV-2 spike for the ferret ACE2. So far, the binding of SARS-CoV-2 to another viral entry receptor than ACE2 cannot be excluded. Very recently, it was reported that the enhanced human spreading of SARS-CoV-2 compared to SARS-CoV could possibly be explained by the presence of a polybasic furin type cleavage site at the S1/S2 junction in the SARS-CoV-2 spike, which is not found in SARS-CoV and that neuropilin-1 (NRP1) known to bind furin-cleaved substrates could be an entry cofactor that potentiates SARS-CoV-2 infectivity (53).

Obviously, not all the species expressing an ACE2 predicted to bind SARS-CoV-2 are expected to be susceptible to infection by SARS-CoV-2. *In silico* studies focused on ACE2 protein polymorphism among species together with focused attention on amino acids expected to play a crucial role in the viral spike binding are suitable to predict ACE2 proteins susceptible to bind SARS-CoV-2 and can provide important clues regarding possible intermediate hosts or simply susceptible hosts. The ACE2 protein should contain amino acids essential for the viral spike binding and variants of ACE2 that lack such amino acids are not likely to allow virus binding. An impressive study combining phylogenetic analysis and critical site marking to predict the utilizing capability of ACE2 recently reported by Qiu et al. (46) compared the ACE2 sequences from 250 species with a specific focus on T20, K31, Y41, K68, Y83, S218, A246, K353, D355, R357, M383, P426, T593, N636, A714, R716, and A774 and concluded that SARS-CoV-2 might bind *M. javanica* (pangolin), *F. catus* (cat), *Bos taurus* (cow), *Bubalus* (buffalo), *Capra hircus* (goat), *Ovis aries* (sheep), and *Columba livia* (pigeon) ACE2 but not (*M. musculus*) murine ACE2. They also suggested to pay attention to *Protobothrops mucrosquamatus* (pallas pit viper), a common snake living in the Hubei Province of China. In their study, Luan et al. (45), investigated 42 mammalian ACE2 proteins from the wild animal protection list of Hubei Province. The authors focused on key amino acids K31, E35, D38, M82, and K353. According to their predictions, they considered that beside humans, the mammals whose ACE2 could bind to the S1 protein of SARSCoV-2 are bats (*Rhinolophus macrotis, Rhinolophus sinicus, Rhinolophus pearsonii, Pteropus vampyrus*, and *Rousettus leschenaultii*), pangolin (*Manis javanica*), palm civet (*Paguma larvata*), monkeys (*Macaca mulatta, Pan troglodytes, Pongo abelii, Papio Anubis*, and *Callithrix jacchus*), cat (*Felis catus*), dog (*Canis lupus familiaris*), ferret (*Mustela putorius furo*), and pig (*Sus scrofa domesticus*), among others (*Rhinopithecus roxellana, Mustela erminea, Sus scrofa, Equus caballus, Bos taurus, Ovis aries, Oryctolagus cuniculus, Vulpes, Phodopus campbelli, Mesocricetus auratus, Heterocephalus glaber, Ictidomys tridecemlineatus*, and *Cricetulus griseus*). The mammals whose ACE2 appeared unable to bind the S1 protein of SARS-CoV-2 included *Rhinolophus ferrumequinum* bats, rat (*Rattus norvegicus*), mouse (*Mus musculus*), camel (*Camelus dromedarius*), and others (*Procyon lotor, Ornithorhynchus anatinus, Loxodonta africana, Erinaceus europaeus, Nyctereutes procyonoides, Suricata suricatta, Dipodomys ordii*, and *Cavia porcellus*). They draw particular attention to the N82 amino acid in the ACE2 protein. Another study by Liu et al. (44), based on prediction of interactions between the S1 protein of SARS-CoV-2 and ACE2, that investigated monkey (*Gorilla, Macaca*), bat (*Rhinolophus sinicus*; *Rhinolophus pearsonii*), pangolin (*Manis javanica*), snake (*Ophiophagus hannah*), turtles (*Chrysemys picta bellii, Chelonia mydas*, and *Pelodiscus. sinensis*), and others (dog, cat, mouse), stressed a possible role as intermediate host animal reservoir for turtles. This study, which focused on positions T27, F28, D30, K31, H34, D38, Y41, Q42, M82, E329, K353, G354, D355, and R357, indicated that mouse and dog ACE2 showed multiple substitutions (>5) among the 14 amino acids that retained their attention, an observation in agreement with the relative resistance of these species to infection by SARS-CoV-2. They suggested K31, Y41, and K353 to be key amino acids for viral spike binding. In recent weeks, several *in silico* studies aimed at finding an intermediate host have been published. Luan et al. (45) ruled out turtle and snake from the potential host list of SARS-CoV-2 and suggested that pangolin ACE2 was predicted to recognize SARS-CoV-2 less efficiently because it only preserved 14 of 20 critical amino acids they investigated, but found that primates, Bovidae, Cricetidae, and Cetacea (*Neophocaena asiaeorientalis asiaeorientalis*, found in the Yangtze River near Wuhan), are capable to recognize the RDB in S1 of SARS-CoV-2. A very elegant work by Damas et al. (54) scored 25 amino acids considered by this team as important for interaction between SARS-CoV-2 spike and ACE2 and they identified possible interaction for 252 mammal species, 72 birds, 65 fishes, 17 reptiles, and 4 amphibian ACE2 orthologs. It is worth noting that species scoring very low in Damas' study included the Chinese pangolin, Sunda pangolin, and white-bellied pangolin. Among Carnivora, 9/43 had the highest score including the domestic cat. Similar approaches that indicate a broad range of possible animal targets for SARS-CoV-2 are currently under evaluation for publication (44, 55). We can therefore also retain from these studies that, according to *in silico* analyses, numerous species are potentially susceptible to infection by SARS-CoV-2. This is a strong argument in favor of the virus circulation model in which there is not a single intermediate host but many susceptible species (33).

Although the *in silico* studies have the advantage of being easy to perform and to allow a quick investigation of the probability of SARS-CoV-2 infection for a large number of species, this strategy has its limits, and possible receptor binding does not necessarily mean successful replication in host. Once in the host, the virus should counteract the cell restriction factors and antiviral immune defense. Nothing can replace *in vitro* and *in vivo* experimentation. *In vitro*, SARS-CoV-2 was found to be able to infect and replicate on human Calu3 and Caco2 cell lines, VeroE6 and FRhK4 from non-human primate cell lines, LLCMK2 (monkey), RK-13 (Rabbit), PK-15 (pig), and CRFK (cat) cell lines (56). Interesting observations reported online (not peer reviewed) indicate that multiple ACE2 orthologs, human (*H. sapiens*), rhesus monkey (*M. mulatta*), dog (*C. lupus familiaris*), cat (*F. catus*), rabbit (*O. cuniculus*), and pangolin, can serve as receptors for SARS-CoV-2 when transiently expressed in 293T cells, whereas rat (*R. norvegicus*) ACE2 does not (8).

Even when cells from a species are susceptible to SARS-CoV-2, this does not always translate into disease. Although it is more fastidious work than *in silico* and *in vitro* approach, evidence supporting that a species is susceptible to SARS-CoV-2 and can develop COVID-19-like symptoms can only be defined after *in vivo* infection. Interestingly golden Syrian hamster (*M. auratus*) and Chinese hamster (*C. griseus*) are known as animal models for SARS-CoV (57, 58). More recently, the golden Syrian hamster has been established as a model to study the transmission of SARS-CoV-2 and the pathogenesis of COVID-19 (59, 60). Monkeys (*M. mulatta, Macaca fascicularis*, and *Chlorocebus aethiops*) were also found to be animal models for SARS-CoV with reports of pneumonitis in infected monkeys (61, 62). With SARS-CoV-2, monkeys (*M. mulatta* and *M. fascicularis*) were found to be susceptible to the virus and develop mild disease COVID-19-like signs after infection (63, 64). Ferrets (*M. putorius furo*) were also used as an animal model for SARS-CoV and showed productive infection (65, 66). This species also was found to be susceptible to SARS-CoV-2 and develop mild disease COVID-19-like signs after infection (49**?** –51). It was previously reported that young inbred mice supported SARS-CoV viral replication but failed to show clinical signs of disease (67, 68). Although mouse (*M. musculus*) ACE2 was considered unable to bind SARS-CoV-2 spike (35) and unable to support SARS-CoV-2 replication and disease development (69), it was reported that *M. musculus* transgenic for the human ACE2 gene are susceptible to infection by SARS-CoV-2 and develop mild disease COVID-19-like signs after viral exposure (38, 69). The paper recently published by Shi et al. (52) describes the investigation of the *in vivo* susceptibility of animals to replicate SARS-CoV-2. The authors reported that the virus replicated poorly in dogs, pigs, chickens, and ducks but efficiently infected ferrets and cats. In addition, these authors found that the virus can be transmitted from cat to cat through respiratory droplets. This result agrees with the report of experimental cat-to-cat transmission of SARS-CoV-2 (70) and human-to-cat transmission of SARS-CoV-2 (71). The accidental transmission of SARS-CoV-2 to tigers and lions at the Bronx Zoo (72) and minks (73) was also reported. Finally, Schlottau et al. (50) reported that pig and chickens were not susceptible to SARS-CoV-2 infection, whereas efficient virus replication was found in ferrets and fruit bats. The results obtained in our *in silico* study were compared with those of *in vivo* infection reported by different research teams (Table 2), and we observed a good match between the two experimental approaches.

**Table 2:** Correlation between *in silico* ACE2 binding prediction and *in vivo* SARS-CoV-2 infection.

**Species**	**Probability of SARS-CoV-2 binding to ACE2 (*in silico* prediction and score)**	***In vivo* SARS-CoV-2 replication (G clade virus)**	**Bibliographical references for the *in vivo* experimental infections**	**Agreement between *in silico* prediction and *in vivo* data**
Human	Yes (Score 5)^*^	COVID-19 outbreak	(1–3)	Reference model
Monkey	Yes (Score 5)	Susceptible (COVID-19-like signs)	(63, 64)	Yes
Civet	No (Score 2)	Not tested		Not applicable
Cat	Yes (Score 4)	Susceptible to infection	(52, 70)	Yes
Ferret	Yes (Score 3)	Susceptible (COVID-19-like signs)	(49, 50, 52)	Yes
Pig	Yes (Score 3)	Susceptible, yet the virus replicates poorly	(50, 52)	Yes
Boar	Yes (Score 3)	Not tested		Not applicable
Bat	Yes (Score 3)	Susceptible to infection	(50)	Yes
Pangolin	Yes (Score 4)	Not tested		Not applicable
Mouse	No (Score 1)	Resistant to infection (hACE2 humanized mice are susceptible to infection and show interstitial pneumonia)	(38, 69, 74)	Yes
Rat	No (Score 2)	Not tested		Not applicable
Hen	No (Score 2)	Not tested		Not applicable
Turtle	Yes (Score 3)	Not tested		Not applicable
Frog	No (Score 1)	Not tested		Not applicable
Snake	No (Score 2)	Not tested		Not applicable

* *Score 5: K31, Y41, N90, K353 (+4/4) and no mutation in regions 31–41, 82–93, and 353–358 with respect to the human ACE2 (hACE2) sequence (+1); Score 4: A change for one of the positions K31, Y41,N90, or K353 (+3/4), and no mutation in regions 31–41, 82–93, and/or 353–358 (+1) or K31, Y41, N90, and K353 (+4/4), and mutations in regions 31–41, 82–93, and/or 353–358 (0/1); Score 3: two variants at positions K31, Y41,N90, or K353 (+2/4) and no mutation in regions 31–41, 82–93, and 353–358 (+1), or a change for one of the positions K31, Y41, N90, or K353 (+3/4) and mutations in regions 31–41, 82–93, and/or 353–358 (0/1); Score 2: three variants at positions K31, Y41,N90, or K353 (+1/4) and no mutation in regions 31–41, 82–93, and/or 353–358 (+1) or two variants at positions K31, Y41, N90, or K353 (+2/4) and mutations in regions 31–41, 82–93, and/or 353–358 (0/1); Score 1: three variants at positions K31, Y41, N90, or K353 (+1/4) and mutations in regions 31–41, 82–93, and/or 353–358 (0/1). Arbitrary cut off: it was considered that a score ≥3 is predictive of attachment of the viral spike to ACE2 that can lead to infection*.

Finally, if the absence of productive infection in animal models makes it possible to exclude certain species from the dynamics of transmission of SARS-CoV-2 to humans, the finding of productive infection provides little information on the origin of the human COVID-19 epidemic and pandemic. Human epidemic can only occur when there is a contact between human and an infected species, when this pathogen is compatible with human, and when human-to-human urban cycle is possible. The spillover model of virus transmission theorizes that the virus is developing into an epizootic stage in an animal population, reaching the threshold requirement for interspecies transmission (39). Thus, based on this model, identifying an animal reservoir appears to be essential to eradicate the disease by eliminating the infected animal host species. However, what we observe from the increasing number of reports aimed at identifying an animal reservoir is that numerous animal species are susceptible to SARS-CoV-2 and that no epizootics was reported with a SARS-CoV-2-like ancestral virus. This is the reason why it was recently suggested to consider a new model, the circulation model, which assumes that there is a broad circulation of virus in different species, and no requirement for zoonotic pressure or epizootic episode prior to the COVID-19 emergence in human (33). According to this new model, if the SARS-CoV-2-like ancestral virus can meet a host, if the virus spike RBD can bind ACE2 molecule even at low affinity, and if the target cells can be productively infected, then the adaptation to the host simply undergoes a quasispecies evolution process. So the scenario that can be suggested here is that the virus was circulating in many species, that following contact between one of these species and humans, a SARS-CoV-2-like virus came into contact with the ACE2 protein at the surface of human lung epithelial cells allowing infection to occur. ACE2 (100-kDa type I cell-surface glycoprotein of 805 amino acids) is expressed on both type I and type II alveolar epithelial lung cells as well as epithelial cells of oral mucosa, enterocytes of the small intestine, and arterial and venous endothelial cells contributing to the COVID-19 disease (38, 75–78). Currently, SARS-CoV-2 is expected to undergo a quasispecies evolution process generating post-infection mutations under host-driven positive selection pressure (32, 79–83).

In conclusion, our results suggest that species carrying a sequence with K31, Y41, N90, and K353 are likely to be susceptible to infection by SARS-CoV-2 (including *H. sapiens, M. mulatta, F. catus, R. sinicus, M. javanica*, and *P. sinensis*) while others should be less susceptible or resistant to infection, except if the virus adapts a second receptor for cellular binding and entry. The combination of 3-D structure analysis and electrostatic potential surface indicated that the substitution of human ACE2 regions 30–41, 82–93, and 353–358 by the corresponding regions from *R. sinicus, M. musculus*, and *X. tropicalis* species did not significantly change the 3-D structure of ACE2 but slightly modified the electrostatic potential surface of the molecule. These modifications are likely to be sufficient to alter the interaction of SARS-CoV-2 spike with the variants ACE2. The K31 and K353 in the α-helical bundle of the ACE2 interface need to be accommodated in a largely hydrophobic environment to allow interaction with the viral spike RBD (9). The crystal structure analysis of ACE2 also suggested the presence of several hinge regions and N-glycosylations (9, 84), including the glycosylation of N90 considered essential for SARS-CoV-2 binding. The ACE2 NxT/S consensus N-glycosylation motif (54, 85) is altered in 9 out of 19 bat species tested in this study (Figure 1). It is also absent on ACE2 from a number of species such as mouse or rat, which are considered resistant to infection, while it is present in species that have been shown to be susceptible to SARS-CoV-2 such as human, monkey, or cat (Figure 2). This highlights the *in silico* approach as a simple screening tool to identify species susceptible to SARS-CoV-2 in a given ecosystem (74, 86–88). SARS-CoV-2 infection was recently reported in mink farms and there is evidence that employees were infected with SARS-CoV-2 after minks became infected, suggesting that mink farms might become a reservoir for future spillover of SARS-CoV-2 to humans (73). We have aligned the mink ACE2 partial sequence available from GenBank (GenBank CCP86723.1) with the human ACE2 and observed that the mink ACE2 carries the K353 amino acid, but it was not possible to compare the other amino acids (K31, Y41, and N90) important for SARS-CoV-2 binding because the N-terminal part (1–318) of the protein is missing (data not shown). Facing the SARS-CoV-2 epidemic in minks, in the last few days, the Danish Government announced the culling of 17 million minks in rearing after researchers in Denmark have identified some 170 mutations (including a Y453F mutation in the viral spike) in samples from 40 mink farms and the report of mink-specific mutations of SARS-CoV-2 found in humans (89). The rationale behind this decision is the risk that these mutations might allow the virus to spread more easily among people, make it more deadly, and negatively impact the deployment of anti-COVID-19 vaccines. However, there is little evidence that these mutations are of particular concern; the real drivers of epidemics and pandemics are human activities, and trying to eradicate all supposed animal sources of infection is probably more fearful than rational (90).

## Data Availability Statement

The sequence data presented in this study can be found in the online ncbi database repositories. All sequences can by found at https://www.ncbi.nlm.nih.gov/genbank and the accession numbers are indicated in the Materials and Methods section.

## Author Contributions

CD performed the Clustal Omega analysis, designed the figures, and wrote the paper. LP performed the 3-D analysis. IO worked on the art design of figures. DR obtained the funding and supervised the study. All authors reviewed and approved the final version of the manuscript. All authors contributed to conceive the manuscript.

## Conflict of Interest

The authors declare that the research was conducted in the absence of any commercial or financial relationships that could be construed as a potential conflict of interest.
